# Identification and Expression Profiling of Two Saudi Arabia Catalase Genes from Wheat and Barley in Response to Abiotic and Hormonal Stresses

**DOI:** 10.3390/antiox11112208

**Published:** 2022-11-08

**Authors:** Mouna Ghorbel, Malek Besbes, Najla Haddaji, Nouha Bouali, Faiçal Brini

**Affiliations:** 1 Biology Department, Faculty of Science, University of Hail, Ha’il 2440, Saudi Arabia; 2 Laboratory of Biotechnology and Plant Improvement, Center of Biotechnology of Sfax, Sfax 3018, Tunisia

**Keywords:** antioxidant enzymes, bioinformatic analysis, catalase, oxidative stress, peroxisomal targeting signal, 2D structure

## Abstract

Catalase is a crucial enzyme in antioxidant defense systems protecting eukaryotes from oxidative stress. These proteins are present in almost all living organisms and play important roles in controlling plant responses to biotic and abiotic stresses by catalyzing the decomposition of H_2_O_2_. Despite their importance, little is known about their expression in the majority of monocotyledonous species. Here, we isolated and characterized two novel catalase genes from *Triticum turgidum* and *Hordeum vulgare*, designated as TtCAT1 and HvCAT1, respectively. Phylogenetic analysis revealed that TtCAT1 and HvCAT1 presented 492 aa and shared an important identity with other catalase proteins belonging to subfamily 1. Using bioinformatic analysis, we predicted the 3D structure models of TtCAT1 and HvCAT1. Interestingly, analysis showed that the novel catalases harbor a peroxisomal targeting signal (PTS1) located at their C-terminus portion, as shown for other catalase proteins. In addition, this motif is responsible for the in silico peroxisomal localization of both proteins. Finally, RT-qPCR analysis showed that TtCAT1 and HvCAT1 are highly expressed in leaves in normal conditions but faintly in roots. Moreover, both genes are upregulated after the application of different stresses such as salt, osmotic, cold, heavy metal, and hormonal stresses. The positive responses of TtCAT1 and HvCAT1 to the various stimuli suggested that these proteins can help to protect both species against environmental stresses.

## 1. Introduction

Plants are exposed to many abiotic stresses such as water logging, drought, cold, salinity, and extreme temperatures. These stresses generate reactive oxygen species (ROS) such as superoxide oxygen (O_2_^●−^) and hydrogen peroxide (H_2_O_2_) in plant cells. ROS could be produced in different cell compartments such as chloroplasts, mitochondria, and peroxisomes [1,2]. These particles are important signaling components regulating many biological processes. Moreover, they are toxic and highly reactive at high concentrations [3,4]. In fact, ROS particles are responsible for severe damage to lipids, proteins, and nucleic acid as they impose oxidative stress. To avoid such negative effects on cells, ROS must be transformed into less dangerous forms. Plants dispose an efficient scavenging system with different antioxidant enzymes [5]. Among enzymes, catalases and peroxidases have the most efficient role due to their high affinity for H_2_O_2_ to convert it to oxygen and water [6]. Catalases are powerful antioxidant metalloenzymes located in peroxisomes as shown for many other catalases such as genes isolated from *Triticum durum* [7] and *Triticum monococcum* [8]. This location is controlled by a conserved motif, a peroxisomal targeting signal type 1 (PTS1), located at the C-terminal portion of the protein, which is the most common peroxisome targeting protein in plants [9]. Animal genomes contain a single catalase gene, whereas in plants, this enzyme is decoded by a multigenic family yielding multiple isoenzymes. The number of catalases varies depending on the species, and their expression is regulated according to their tissue/organ distribution and the environmental conditions. So far, different catalase genes have been characterized in various plant species such as *Triticum durum* [7], *Triticum monococcum* [8], *Gossypium hirsutum* L. and *G. barbadense* L. [10], *Cucumis sativus* L. [11], rice [12], *Triticum aestivum* [13], and *Brassica napus* [14]. Recently, four different catalase genes were identified from barley. CAT2 and CAT4 were activated in response to drought stress [15]. Catalase genes are involved in plants’ response to both abiotic and biotic stress [6,10,16]. In *Arabidopsis*, three isoforms were identified and are regulated in response to the developmental process as well as various abiotic stresses [17,18]. Expression analysis showed that the catalase gene is implicated in plants’ response to infection with the *V. dahlia* Kleb pathogen in *Gossypium hirsutum* L. and *G. barbadense* L. plants [10].

To our knowledge, little is known about protein characterization in wheat and barley species cultivated in Saudi Arabia. Thus, we isolated the full length of HvCAT1 and TtCAT1 cDNA sequences from *Triticum turgidum* and *Hordeum vulgare*, respectively. The encoding proteins are very similar to wheat catalases TdCAT1 (*Triticum* durum, AKC00864.1), TaCAT1 (*Triticum aestivum*, NP_001392633.1), and TmCAT1 (*Triticum monococcum*, QBZ38484.1), suggesting structure conservation of these proteins among species and varieties. We determined their three-dimensional structure, functional domains, and sequence homology with other catalases. In addition, we studied the expression pattern and the role of TtCAT1 and HvCAT1 in Saudi varieties in response to various environmental stresses. These results provide a new insight into the understanding of the physiological functions of catalase genes in monocotyledons.

## 2. Materials and Methods

### 2.1. Plant Material

Seeds of Saudi Arabia varieties of *T. turgidum* subsp durum (cv. Waha) and *H. vulgare* were collected from private fields in Al-Khuttah, north-west of Hai`l, Saudi Arabia. Before incubation, almost 50 seeds were treated for 15–20 min with 30 mL of a 0.5% sodium hypochlorite solution and then washed four times with 50 mL of sterile water. Seed germination was performed in Petri dishes (11 cm wide, 11 cm long, and 2.5 cm high) containing a sheet of Whatman filter paper and a piece of sponge (to maintain moisture) at 25 °C ± 2 °C under photosynthetically active radiation of 280 μmol m^−2^ s^−1^ with 16/8 h light/dark conditions, and the light conditions were fixed at 250 μmol m^−2^ s^−1^. Seeds were then placed in a greenhouse. Different stress treatments were then applied to the seeds 10 days after incubation. In this study, ten treatments were applied including a control (distilled water), 10% PEG, 200 mM of mannitol, 150 mM of NaCl, heat (42 °C), and 5 mM of each phytohormone (SA and ABA). Each treatment was replicated three times. Finally, shoots and roots were harvested, immediately frozen in liquid nitrogen, and stored at −80°C.

### 2.2. Isolation and Molecular Cloning of TtCAT1 and HvCAT1 cDNA

Before starting, sequence alignment of different full sequences of monocotyledonous CAT1 genes was performed, and primers corresponding to the 5′ and 3′ UTR regions were designed to be used to amplify TtCAT1 and HvCAT1.

Total RNAs were isolated from the leaves of *T. turgidum* and *H. vulgare* plants subjected to 150 mM of NaCl for 24 h using the TRIzol reagent (Invitrogen) according to the manufacturer’s protocol. In order to eliminate the lasting genomic DNA, the extracted RNA was treated with RNase-free DNase. Then, 2 μg of total RNA were used to synthesize the first-strand cDNA using M-MLV reverse transcriptase (Invitrogen) as detailed in the manufacturer’s protocol. Newly synthetized cDNAs were then used to amplify the TtCAT and HvCAT genes. After cloning and sequencing of the fragments, both full-length cDNAs were amplified using two specific primers, CAT1_Fr (5′-ATGGCATCTTCCAAGAGCAG-3′) and CAT1_Rv (5′-TCAAGGGTGAGGACGCGAA-3′). Specific primers were designed using the TdCAT1 sequence (accession no. KP696753) [7] and the HvCAT1 sequence (accession number XP_044983038.1). The PCR conditions were conducted as follows: an initial denaturation at 94 °C for 5 min followed by 30 cycles comprising a first step at 94 °C for 30 s, an annealing step at 57 °C for 30 s, an elongation step at 72 °C for 90 s, and a final 10 min extension at 72 °C. PCR products were separated by 1% agarose gel, then gel purified, cloned in the pGEM-T Easy vector, sequenced using the T7 and SP6 primers by the ABI PRISM automated sequencer, and then submitted to GenBank.

### 2.3. Bioinformatic Analysis of the Sequence of Catalase 1 Genes

Prediction of the physicochemical parameters of the TtCAT1 and HvCAT1 proteins was carried out as follows: the theoretical molecular weight (MW), the aliphatic index (AI), the isoelectric point (pI), and the grand average of hydropathy (GRAVY) were predicted using the ProtParam tool (http://web.expasy.org/protparam/; accessed on 10 May 2022). Signal peptide prediction sequences’ presence and the location of their cleavage sites were predicted by the SignalP 5.0 Server (http://www.cbs.dtu.dk/services/SignalP/; accessed on 10 May 2022). The InterPro program was used to identify the functional domains of TtCAT1 and HvCAT1. Multiple sequence alignment of the CAT1 protein sequences from different plants was performed by ClustalX implemented in the MEGA7.0 software [19]. The phylogenetic tree was constructed using the maximum likelihood method and 1000 bootstrap replicates using the MEGA7.0 software. Secondary structures of proteins were predicted by SOPMA (https://npsa-prabi. ibcp.fr/cgi-bin/npsa_automat.pl?page=npsa_sopma.html [20]; accessed on April 12, 2022), whereas 3D structures were predicted using enzymol (http://www.sbg.bio.ic.ac.uk/~ezmol/cgi-bin/loadingPage.cgi, [21] accessed on 12 May 2022) and SWISS-MODEL (https://swissmodel.expasy.org/; accessed on 12 May 2022). Cation binding domains were investigated using the UniProt database [22], the SUPFAM database (http://supfam.org/SUPERFAMILY/cgi--bin/align.cgi (accessed on 14 July 2022) [23], and the SWISS-MODEL database, (https://swissmodel.expasy.org/interactive/T5XR77/models/ (accessed on 14 May 2022) [24] for the identification of Mn^2+^/Mg^2+^, Ca^2+^, Zn^2+^/Cu^2+^, and Fe^2+^ binding domains, respectively.

The presence of conserved CaMBD was identified using the Calmodulin target database (http://calcium.uhnres.utoronto.ca/ctdb/no_flash.htm, [25] accessed on 23 July 2022). Cation binding domains were investigated using the UniProt database [22] and the SUPFAM database (http://supfam.org/SUPERFAMILY/cgi-bin/align.cgi; accessed on 13 May 2022) [26] for the identification of Mn^2+^/Mg^2+^, Ca^2+^, and Zn^2+^/Cu^2+^ binding domains, and the SWISS-MODEL database (https://swissmodel.expasy.org/interactive/T5XR77/models/; accessed on 22 May 2022) [27] was used for the identification of Fe^2+^ binding domains. Subcellular localization of the deduced proteins was predicted using the CELLO2GO web server (http://cello.life.nctu.edu.tw/cello2go/; accessed on 13 May 2022) [28], WoLF PSORT (http://wolfpsort.seq.cbrc.jp/) [29], and EuLoc (http://euloc.mbc.nctu.edu.tw/; accessed on 13 May 2022) [30].

### 2.4. Quantitative Real-Time Polymerase Chain Reaction (qRT-PCR)

To evaluate the expression profiles of TtCAT1 and HvCAT1, *qRT-PCR* was used. Thus, ten days after seed germination, stress treatments were applied. The seedlings were transferred to various solutions: 150 mM of NaCl, 150 mM of sorbitol, 150 mM of mannitol, 15% PEG (*w/v*), 4 °C for 4 h, heavy metals (100 μM of CdCl_2_/AlCl_3_/CuCl_2_), and hormones (5 mM of ABA and SA). Control seedlings were kept in media without stress. Samples were collected after 24, 48, and 96 h of the stress treatments. Each tissue sample (roots and leaves) was collected and pooled together from four plants, immediately frozen in liquid nitrogen, and stored at −80 °C. For RNA extraction, we used the TRIzol method (Invitrogen) using the M-MLV reverse transcriptase for 1 h at 37 °C. The cDNA was synthetized using the oligo-dT (18mer) primer.

For expression analysis, the primers were designed using the Primer 3 software for TtCAT1, HvCAT1, and actin genes using the following primers: qTtCAT1-F: 5′- TCTTCTCCTACTCCGACACG-3′ and qTtCAT1-R: 5′-AGGGGAAGTAGTCGACCTCC-3′; qHvCAT1-F: 5′-ACTTGATGCTCCCCGTGAATG-3′ and qHvCAT1R: 5′- GAGGCATAGGGTATTTTCA-3′; and qAct-F: 5′-CTGACGGTGAGGACATCCAGCCCCTTG- 3′ and qAct-R: 5′-GCACGGCCTGAATTGCGACGTACATGG-3′. Real-time PCR (RT-PCR) was performed in 96-well plates with the CFX 96 Touch TM Real-Time PCR System (Biorad) using the SYBR@Select Master Mix for CFX (Applied Biosystems). The amplification reactions were performed in 10 μL final volumes containing 5 μL of 2 × SYBR @ Select Master Mix, 0.5 μL of each primer (10 μM), 1 μL of RNase-free water, and 3 μL of cDNA (40 ng of cDNA). The reaction consisted of an initial denaturation at 94 °C for 10 min followed by 45 cycles at 94 °C for 10 s, 60 °C for 10 s, and 72 °C for 15 s, then a melting curve (5 s at 95 °C, 1 min at 65 °C, and 5 min with temperature increasing from 65 to 97 °C). The relative expression was quantified using the comparative CT method with the actin gene as an internal expression standard [31]. The relative expression was determined using the formula 2−ΔΔCT. Three biological repetitions were performed for each experimental condition, with three technical repetitions for each sample.

### 2.5. CAT Activity Assay

To analyze the quantitative change in catalase activity in wheat and barley after stress application for 24 h, leaves were sampled as above. For salt stress, controls were irrigated with sterile distilled water, whereas stressed plants were irrigated with 150 mM of an NaCl solution and 15% PEG (*w*/*v*). The frozen leaves of 0.2 g were homogenized in a mortar and pestle with 2.0 mL of ice-cold sodium phosphate buffer (25 mM, pH 7.0) containing 1% (*w/v*) soluble polyvinylpyrrolidine (PVP). The supernatant was centrifuged at 4000 g for 20 min at 4 °C. The soluble protein content was determined by the Coomassie blue dye binding method with a standard curve of bovine serum albumin [32]. After incubation at 25 °C (for blank control, incubated in boiling water for 10 min), 0.2 mL of supernatant was mixed with 1.5 mL of phosphoric acid buffer (pH 7.8) added with polyvinylpyrrolidone (PVP) and 0.3 mL of 0.1 mol/L H_2_O_2_ in a 10 mL tube, which initiated the reaction. CAT activity was monitored by measuring the decrease in absorbance at 240 nm (corresponding to H_2_O_2_ consumption) against a milligram of protein [33]. The decrease in absorbance was recorded by a spectrophotometer followed by the decomposition of H_2_O_2_ at 240 nm and was measured for a total of 5 min [34]. One unit of enzyme activity (U) was defined as the reduced enzyme quantity per min per g of protein. The enzyme activity was calculated as follows:

$$U=\frac{\Delta A_{240}\times V_{T}}{0.1\times V_{1}\times t\times FW}$$

$$\Delta A_{240}=\frac{A_{S0}-A_{S1}-A_{S2}\cdots }{2}$$

AS0 means the absorbance of the blank control;
and AS2 stand for the absorbance of the samples;
VT means the total volume of the crude enzyme solution (mL);
represents the volume of the detected crude enzyme solution (mL);
FW means the fresh weight of the sample (g), and t means the time from adding H_2_O_2_ to the last time (min).
The activity of the catalase was calculated by the activity level of inoculation minus the level of the control at each corresponding time point.

## 3. Results

### 3.1. Isolation and Sequence Analysis of the TtCAT1 and HvCAT1 Genes

**Figure 1:**
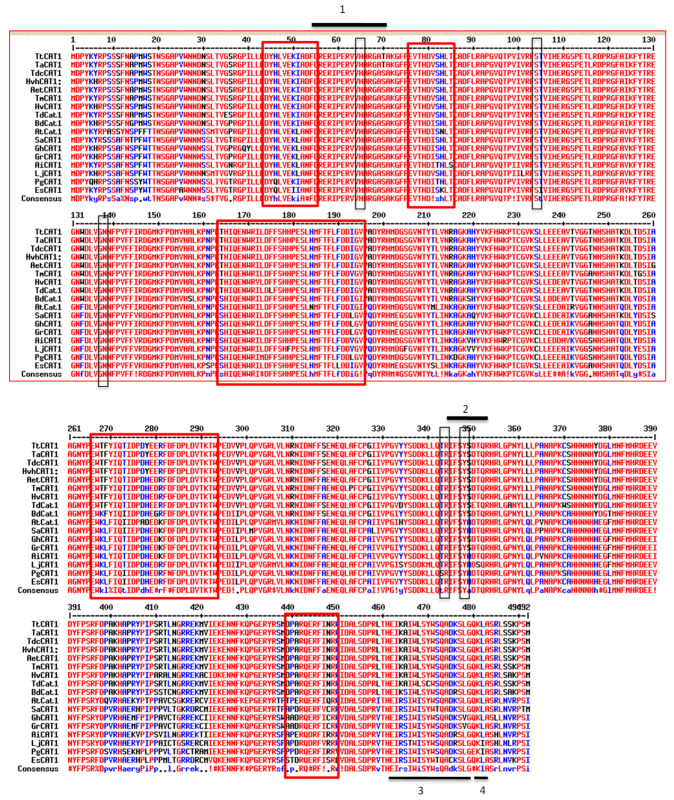
Protein sequence alignment of TtCAT1 (OP434464) and HvCAT1 (OP434465) with other plant catalase proteins identified in *Triticum turgidum* subsp. durum (AKC00864.1), *Triticum aestivum* (NP_001392633.1), *Aegilop stauschii* subsp. tauschii (XP_020164896.1), *Triticum dicoccoides* (XP_037426584.1), *Triticum monococcum* (QBZ38484.1), *Arabidopsis thaliana* (OAO97606.1), *Hordeum vulgare subsp. vulgare* (XP_044983038.1), *Brachypodium dictyoson* (XP_003558892.1), *Panax ginseng* (ABY21704), *Soldanella alpine* (GenBank: CAB16749.1), *Arachis ipaensis* (XP_016167161.1), *Gossypium hirsutum* (P17598), *Lotus japonicus* (AAR84578.1), *Eleutherococcus senticosus* (AHA50082.1), and *Gossypium raimondii* (XP_012464632.1). The conserved His-65; Ser-104; Asn-138; Arg-344; and Tyr- 348 residues implicated in the catalase activity of the proteins are marked with black rectangles. 1: Catalase proximal active site signature (54–70); 2: catalase proximal heme–ligand signature (344–353); 3: calmodulin binding domain (455–481); 4: PTS1 (480–482). Different cation binding domains, Mn^2+^, Fe^2+^, Cu^2+^/Zn^2+^, Ca^2+^, and Mg^2+^, are marked with a red rectangle.

The coding regions of the catalase genes from wheat and barley were amplified by PCR using the total RNA extracted from the leaves of plants of 10 days old and exposed to 150 mM of NaCl for 24 h as a template. The open reading frames of both genes are of 1476 bp nucleotides. Thus, TtCAT1 and HvCAT1 encode for precursor proteins of 492 amino acid residues as revealed by the ProtParam tool (http://web.expasy.org/protparam/; accessed on 15 May 2022), which is the same as that of other identified plant catalases such as durum wheat catalase 1 (TdCAT1, [7]), *Triticum monococcum* (TmCAT1, [8]), and *Arabidopsis* (AtCAT1, [35]). A sequence similarity search showed that HvCAT1 isolated from the Saudi Arabia variety presented 100% identity with the previously isolated sequence (accession number XP_044983038.1), whereas TtCAT1 presented 97.97% identity with TdCAT1 isolated from the Tunisian variety Om Rabiaa (Figure 1). On the other hand, TtCAT1 and TaCAT1 are related and present 100% identity (Figure 1).

The identified catalases present an approximate molecular weight (Mw) of 56.8 kDa and a predicated isoelectric point (pI) of 6.52. In addition, the total number of negatively charged residues is almost the same (59–63 residues) in monocotyledons and dicotyledonous plants (Table 1). In addition, the GRAVY indexes of TtCAT1 and HvCAT1 are negative (−0.595 and −0.591, respectively), which means that these catalase proteins are predicted to be hydrophobic [36,37]. All studied catalase 1 proteins have a negative GRAVY index, which means that all these proteins are hydrophobic (Table 1).

A sequence similarity search showed that TtCAT1 and HvCAT1 share the highest identity with catalases isolated from monocotyledonous species such as wheat catalase TdCAT1 (Table 1). Such results suggest that TtCAT1 and HvCAT1 are similar to TmCAT1 and TdCAT1. TtCAT1 and HvCAT1 have 63 negatively charged residues (Asp + Glu) and 58 positively charged residues (Arg + Lys), which are almost the same among other isolated catalases (Table 1). The TtCAT1 and HvCAT1 sequences were deposited into GenBank with the accession numbers of OP434464 and OP434465, respectively.

The two TtCAT1 and HvCAT1 catalytic active sites as well as heme binding motifs were detected by the ScanProsite tool. Using the same tool, 17 aa (FdReripERvvHarGAT) located at the position of 54–70 was proposed to be a catalase active site signature with the active site located at position 65 for both proteins (H residue). This domain is reported to be involved in catalytic activity, whereas a *catalase proximal heme–ligand signature* (RIFSYSDTQ) is composed of 9 aa and is located at the position of 344–352. This motif is involved in a direct association between the catalase and the heme group (Figure 1; Supplemental Figure S1).

**Figure 2:**
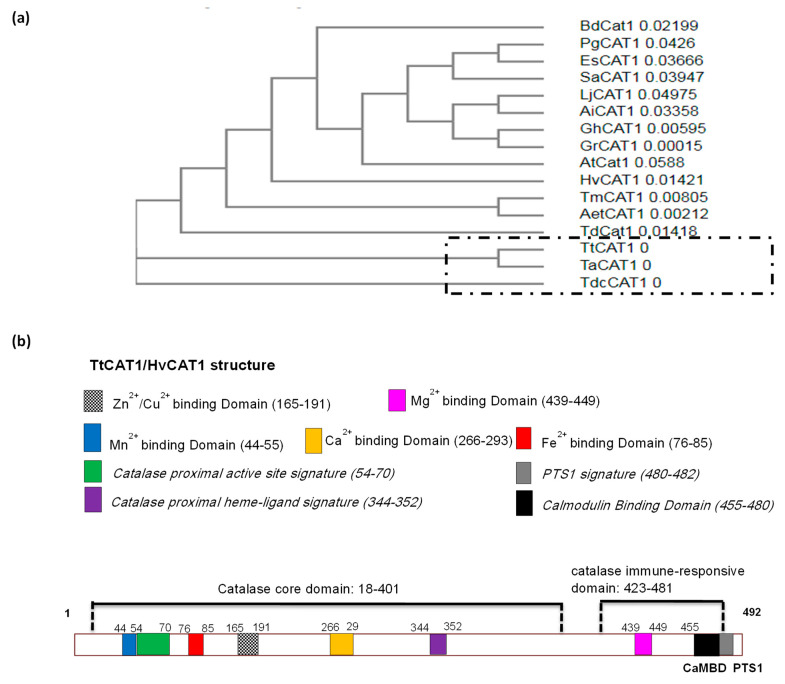
(**a**) Phylogenetic analysis of 16 different catalase proteins, TtCAT1 (OP434464) and HvCAT1 (OP434465), with other plant catalase proteins identified in *Triticum turgidum* subsp. durum (AKC00864.1), *Triticum aestivum* (NP_001392633.1), *Aegilop stauschii* subsp. tauschii (XP_020164896.1), *Triticum dicoccoides* (XP_037426584.1), *Triticum monococcum* (QBZ38484.1), *Arabidopsis thaliana* (OAO97606.1), *Hordeum vulgare subsp. vulgare* (XP_044983038.1), *Brachypodium dictyoson* (XP_003558892.1), *Panax ginseng* (ABY21704), *Soldanella alpine* (GenBank: CAB16749.1), *Arachis ipaensis* (XP_016167161.1), *Gossypium hirsutum* (P17598), *Lotus japonicus* (AAR84578.1), *Eleutherococcus senticosus* (AHA50082.1), and *Gossypium raimondii* (XP_012464632.1). (**b**) Schematic presentation of TtCAT1 and HvCAT1 structures with their potential domains. Both catalases harbor several conserved models such as catalase core domain: 18-401, catalase immune-responsive domain: 423-481, *catalase proximal active site signature: 54-70, catalase proximal heme–ligand signature* 344-353, CaMBD: 455-481, and PTS1 domain: 480–482. Numbers shown represent the position of the amino acid residues of each domain.

A phylogenetic relationship of TtCAT1 and HvCAT1 was constructed using the neighbour-joining method (Clustal Omega software). To make these analyses, different catalases isolated from monocots and dicotyledonous plants were used. As shown in Figure 2a, TtCAT1, isolated from the Waha variety, is closely related to *Triticum aestivum* (TaCAT1) and *Triticum dicoccoide* (TdcCAT1), which belong to class I. As shown in Figure 2a, HvCAT1 is more related to *Brachypodium dictyoson* BdCAT1 and the CAT1 genes isolated from dicotyledons. TtCAT1 and HvCAT1 have no predicted signal peptides at the N-terminal as revealed by SignalP-5.0 (https://services.healthtech.dtu.dk/service.php?SignalP-5.0; accessed on 14 May 2022; data not shown). Furthermore, the InterPro tool showed that TtCAT and HvCAT1 present a catalase core domain (IPR011614) from 18 to 401 residues and a catalase immune-responsive domain (IPR010582) from 423 to 486 (Figure 2b). All these domains are present in monocotyledonous and dicotyledonous plants (Figure 1). Thus, we suggested that the domain structure for catalase proteins is highly conserved in different species. Furthermore, analysis showed the conservation of the five residues that compose the heme pocket: His-65, Ser-l04, Asn-138, Arg-344, and Tyr-348 (Figure 1). These residues form the conserved catalytic residues of the catalases [38]. These residues are conserved in many catalase proteins such as TmCAT1 [8] and TdCAT1 [7]. On the other hand, it has been recently shown that TdCAT1 harbors different cation binding domains located at different parts of the protein. The presence of 2 mM of Mn^2+^, Mg^2+^, Ca^2+^, Fe^2+^, Zn^2+^, or Cu^2+^ stimulated the catalytic activity of TdCAT1 [39]. Sequence alignment of TtCAT1 and HvCAT1 with TdCAT1 revealed that these cation binding domains are conserved in TtCAT1 and HvCAT1 structures (Figure 1). In fact, using the UniProt database (http://www.uniprot.org (accessed on 22 May 2022)), we performed catalase alignment with well-known Mn^2+^ or Mg^2+^ binding proteins. Our analysis showed that TtCAT1 harbors two conserved domains for cation binding. The first domain is located at the N-terminal portion of TtCAT1 (position 44–55; Mn^2+^ binding domain), and the second one is at the C-terminal portion of the protein (position 439–449; Mg^2+^ binding domain) (Figure 1 and 2b). Thus, these domains are highly conserved in different CAT1 proteins identified as previously reported [39]. The other cation motifs (calcium binding domain, copper/zinc binding domain, and iron binding domain) are also conserved (Figure 1; Figure 2b). Using the transmembrane helix prediction (HMMTOP) database, no transmembrane helices were identified in both proteins, indicating that TtCAT1 and HvCAT1 have no function on the membranes in cells. The same result was shown for some other proteins such as PgCAT1 [40]. In addition, the carboxyl terminuses of TtCAT1 and HvCAT1 show a Q-K-L motif similar to other peroxisomal CAT proteins (Figure 1). This motif is known to be crucial for the peroxisomal location of proteins [8]. This motif physically interacts with the receptor protein Pex5p to facilitate the penetration of catalases into peroxisomes [9,41]. On the other hand, it has been shown that the deletion of this motif causes cytoplasmic localization of TdCAT1 and TmCAT1 [8]. To confirm these hypotheses, in silico analyses of TtCAT1 and HvCAT1 using the Cello2Go (http://cello.life.nctu.edu.tw/cello2go/alignment.php; accessed on 15 May 2022) database, the Plant m-Ploc database (http://www.csbio.sjtu.edu.cn/bioinf/plant-multi/; accessed on 25 May 2022), and the Cello V2.5 database (http://cello.life.nctu.edu.tw/cgi/main.cgi; accessed on 22 May 2022) were conducted. Analyses showed that TtCAT1 and HvCAT1 are predominantly peroxisomal proteins with a faint probability to have a mitochondrial and cytoplasmic localization (Supplemental Figure S2).

### 3.2. Structural Homology of TtCAT1 and HvCAT1 Proteins

**Figure 3:**
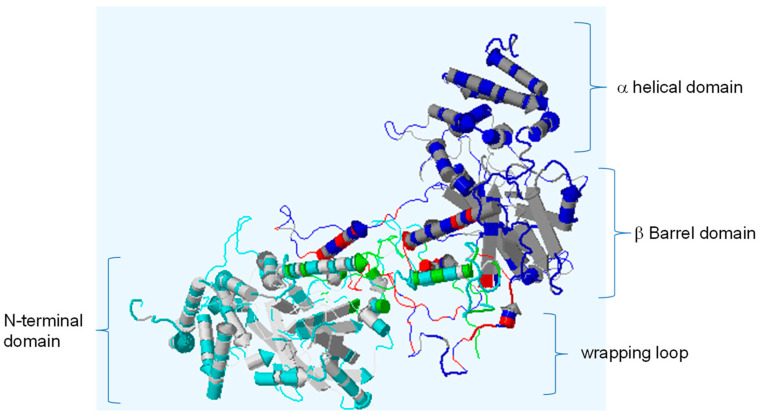
Rocket structure of a monomer of TtCAT1 from durum wheat Waha variety showing four distinct structural regions: an N-terminal arm, the connection domain (wrapping domain), the β-barrel, and the α-helical domain. Residues represented in green represent the five residues composing the heme pocket.

**Figure 4:**
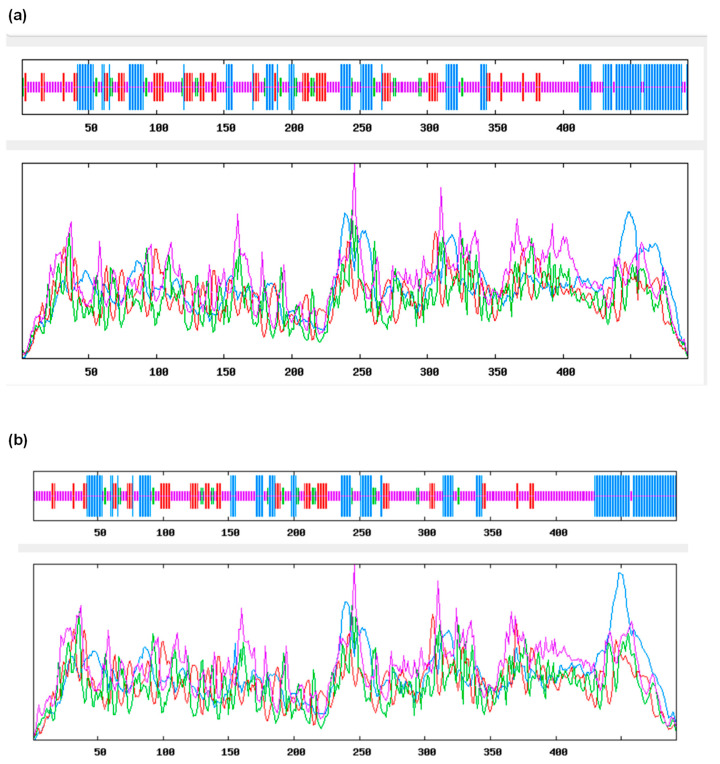
The secondary structures of TtCAT1 (**a**) and HvCAT1 (**b**) as revealed by *SOPMA*. The sheet, turn, helix, and coil are indicated by vertical lines in order from the longest to the shortest.

Using the GenBank Blastp search, we found that TtCAT1 has the closest sequence identity to TaCAT1 and AetCAT1 with 100% and 98.98% identity, respectively. These results are consistent with the phylogenetic relationship of TtCAT1 with other monocotyledonous and dicotyledonous catalase proteins (Figure 2a). To determine the three-dimensional structure of TtCAT1 (100 % similar to HvCAT1), the PDBePISA database was used. To perform this analysis, the crystal structure of the catalase of *Bacillus pumilus* (pdb code: 4QOL) was used as a template [41]. This analysis shows that TtCAT1 is composed of four different subunits (which are the β-barrel domain, the N-terminal arm, the wrapping domain, and the α-helical domain) with the presence of the heme pocket (Figure 3). Interestingly, the C-terminal region (the extra domain) is absent in the TtCAT1 protein. The same structure is also identified in TdCAT1 [7]. The C-terminal domain was identified only in a few typical catalases [7]. In addition, the heme pocket of the TtCAT1 structure is localized between the internal walls of the β-barrel and several helices as previously shown for many catalases [7]. Using the SOPMA online program, we investigated the secondary structure of TtCAT1 and HvCAT1 (Figure 4, Table 2). The analysis revealed that the TtCAT1 structure consists of 140 α-helices, 242 random coils, and 27 β-turns jointed by 83 extended strands (Figure 4). This is very similar to TaCAT1. HvCAT1 presented 136 α-helices, 26 β-turns, 258 random coils, and 72 extended strands (Table 2). These structures are very close to other catalase structures as shown in Table 2.

On the other hand, the Calmodulin target database showed that the TtCAT1 and HvCAT1 sequences harbor a putative calmodulin binding domain (CBD or CaMBD) located at the C-terminal part of the proteins at the residues 464–483 (Figure 1). Such a domain was previously found in many CAT1 proteins such as SPCAT1 [38], TmCAT1 [8], and TdCAT1 [7,39]. In durum wheat, TdCAT1 interacts with CaM in calcium in an independent manner, but the TdCAT1 activity was enhanced in the presence of the CaM/Ca^2+^ complex in calcium in a dependent manner [39]. The CaMBD plays a crucial role during calcium signaling [42]. Taken together, our results suggest that TtCAT1and HvCAT1 are monofunctional heme-containing catalases that possess a putative peroxisomal localization and could be regulated by calmodulin and calcium. All analyses showed that TtCAT1 and HvCAT1 presented four distinct regions typical of monofunctional catalases (Figure 2b). Protein phosphorylation is an important post-translational modification (PTM) controlling crucial cellular processes such as signaling, transport, and nutrient uptake [43]. Thus, TtCAT1 and HvCAT1 structures were analyzed using the NetPhos 3.1 server. TtCAT1 presented 40 putative phosphorylated residues such as S10, S11, S12, T20, T33, T97, S455, T460, S487, and S488 (Supplemental Figure S3a). The same residues were also mapped in TdCAT1 [44]. HvCAT1 presented 37 putative phosphorylated residues such as S10, S11, S15, T33, T97, S455, T460, and S487 (Supplemental Figure S3b). Such results may suggest that catalase proteins could be phosphorylated in cells in response to different stress conditions. Moreover, the GPS-SNO predictor was used to predict the putative nitrosylation residues in the TtCAT1 and HvCAT1 structures. The analysis shows that the TtCAT1 sequence harbors four different nitrosylation sites (C86, C230, C325, and C370), whereas HvCAT1 presented five S-nitrosylation residues (C86, C230, C325, C370 and C421) (Supplemental Figure S4).

### 3.3. Interaction Network of Catalase Proteins

**Figure 5:**
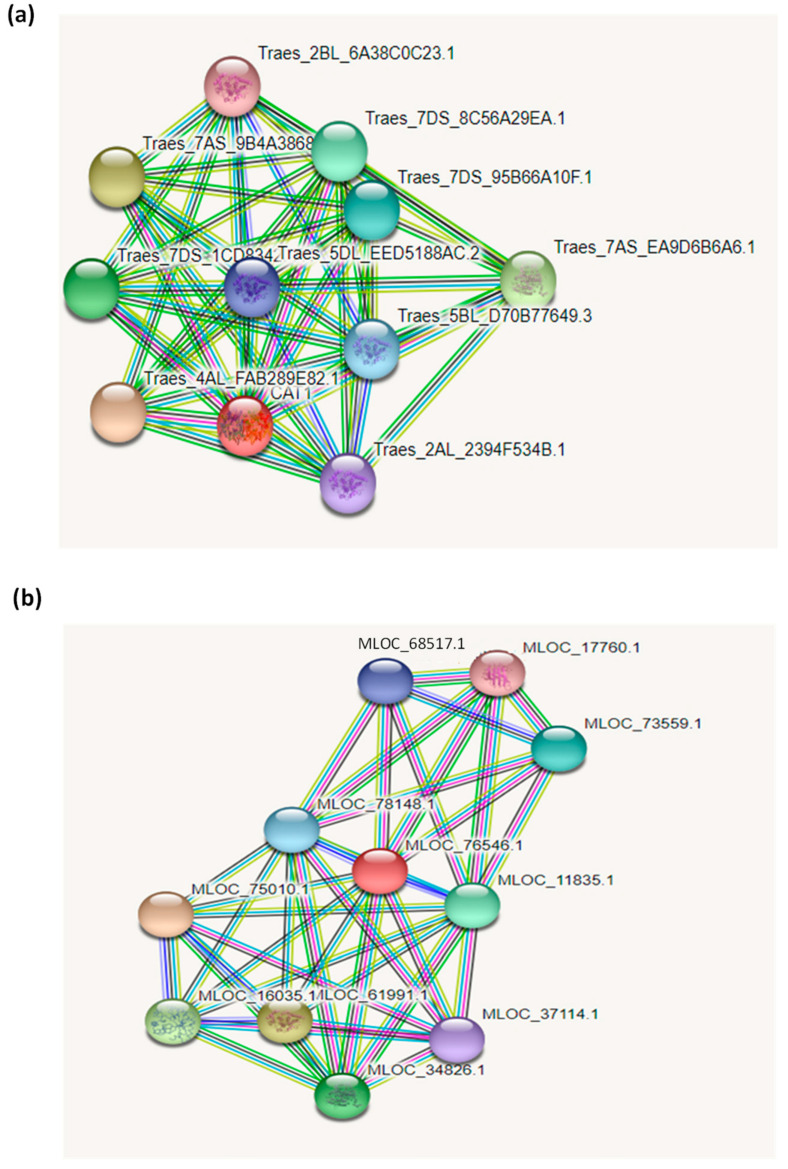
Protein–protein interaction network of the (**a**) TtCAT-1 and (**b**) HvCAT-1 proteins.

The interaction network of the TtCAT1 and HvCAT1 proteins from wheat and barley were constructed based on the interaction relationship of the homologous CAT1 proteins from *Triticum aestivum* and *Hordeum vulgare*, respectively (Figure 5). The interaction network analysis showed that both the TtCAT and HvCAT1 proteins interacted with acyl-coenzyme A oxidase and the FMN hydroxy acid dehydrogenase domain-containing protein (uncharacterized protein). The analysis showed that the TtCAT1 protein interacts with the PKS_ER domain-containing protein (uncharacterized protein). In barley, HvCAT1 interacts with glutathione peroxidase, the TPR_region domain-containing protein (uncharacterized protein), and Zn–Cu superoxide dismutase (Figure 5).

### 3.4. RT-qPCR Analysis of TtCAT1 and HvCAT1 Gene Expressions

**Figure 6:**
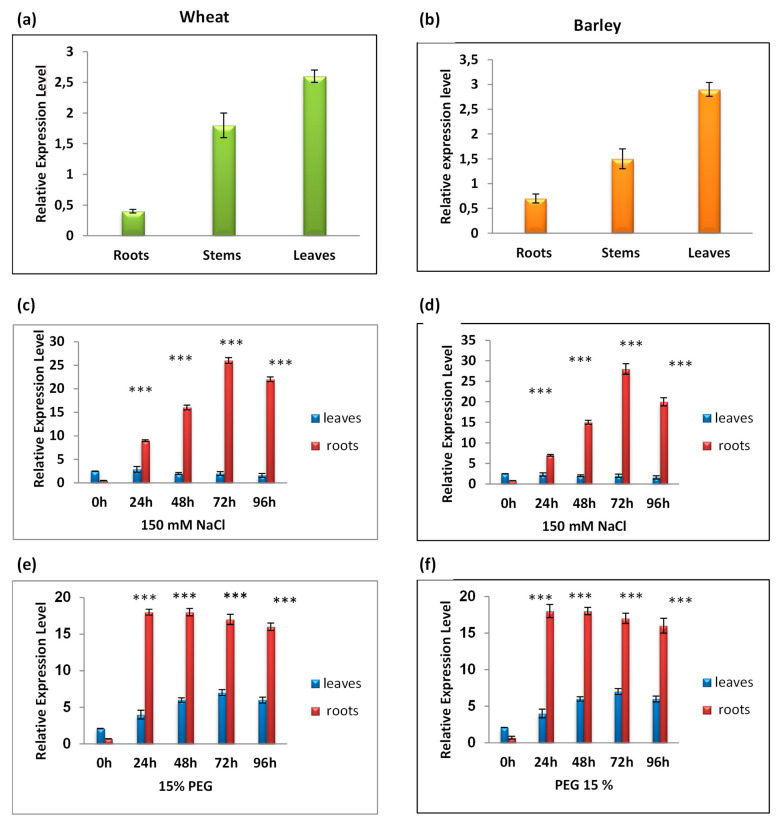
RT-qPCR expression analysis of TtCAT1 and HvCAT1 genes under normal conditions (**a**,**b**) and under different abiotic stresses: 150 mM of NaCl (**c**,**d**) and PEG 15% (**e**,**f**). The red bars represent the expression levels of the CAT-1 gene in wheat, and the blue bars represent the expression level of the CAT-1 gene in barley. *** Indicates values significantly different from the control. Statistical significance was assessed by applying Student’s *t*-test at *p* < 0.01.

The tissue-specific expressions of TtCAT1 and HvCAT1 were conducted using real time qRT-PCR in different tissues of *T. turgidum* sp durum cv. Waha and *Hordeum vulgare*. Under normal conditions, TtCAT1 and HvCAT1 genes were significantly expressed in leaves, but these expressions were very low in roots of both species (Figure 6a,b). In light of these results, we suggest that TtCAT1 and HvCAT1 genes may play fundamental roles in the scavenging of H_2_O_2_ during plant development. Such findings were also shown in other plants such as *Triticum monococcum* [8], *T. durum* cv. Om Rabiaa [7], and *P. ginseng* [41]. The CAT1 gene was significantly expressed in *N. plumbaginifolia* leaves. CAT1 was also expressed with lower levels in the stem and flowers, but it was completely absent in the roots of *N. plumbaginifolia* [45]. In a second step, we investigated the role of TtCAT1 and HvCAT1 in plants’ response to different stress conditions.

As indicated in Figure 6c, the expression of TtCAT1 is strongly induced in roots and reached its maximum accumulation after 72 h of salt stress application. This expression started to decrease after 72 h of stress application, but the expression level was higher than the normal conditions after 96 h of stress application (Figure 6c). In leaves, the expression level of TtCAT1 slightly increased and reached its maximum after 72 h of stress application with a two-fold increase in its expression level (Figure 6c). The same effect was also observed in barley as shown in Figure 6d. In the presence of PEG (15%), the catalase gene expression increased slightly in leaves with a maximum expression after 72 h of stress application, and then it started to decrease (Figure 6e), whereas in roots, the expression level of TtCAT1 increased rapidly and reached its maximum after 24 h of stress application (Figure 6e). The same result was also observed for HvCAT1 (Figure 6f).

**Figure 7:**
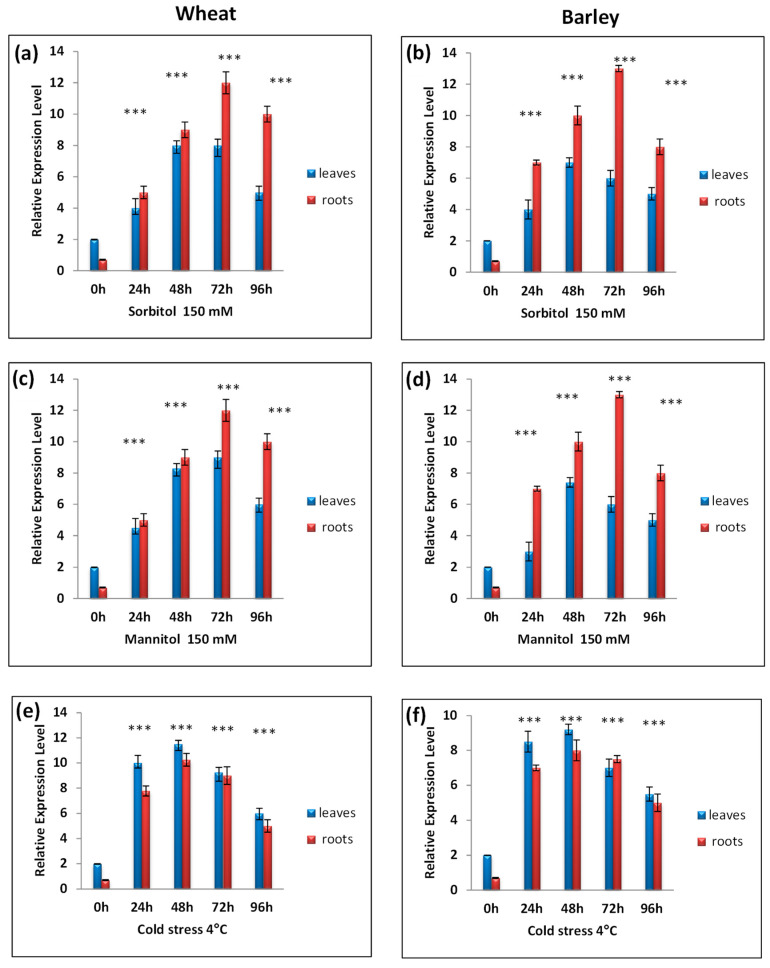
RT-qPCR expression analysis of TtCAT1 and HvCAT1 genes under different abiotic stresses: (**a**,**b**) sorbitol 150 mM, (**c**,**d**) mannitol 150 mM, and (**e**,**f**) cold stress at 4 °C for 4 h in both wheat and barley species. The red bars represent the expression levels of the CAT-1 gene in wheat, and the blue bars represent the expression levels of the CAT-1 gene in barley. *** Indicates values significantly different from the control. Statistical significance was assessed by applying Student’s *t*-test at *p* < 0.01.

The expressions of TtCAT1 and HvCAT1 in response to 150 mM of sorbitol and 150 mM of mannitol were the same as observed in the presence of PEG stress (Figure 7a–d). In wheat, the expression level reached its maximum after 72 h and started to decrease after 96 h. TtCAT1 and HvCAT1 genes were also expressed in response to cold stress (Figure 7ef), with maximum expressions detected after 48 h of stress application for both species (Figure 7ef).

**Figure 8:**
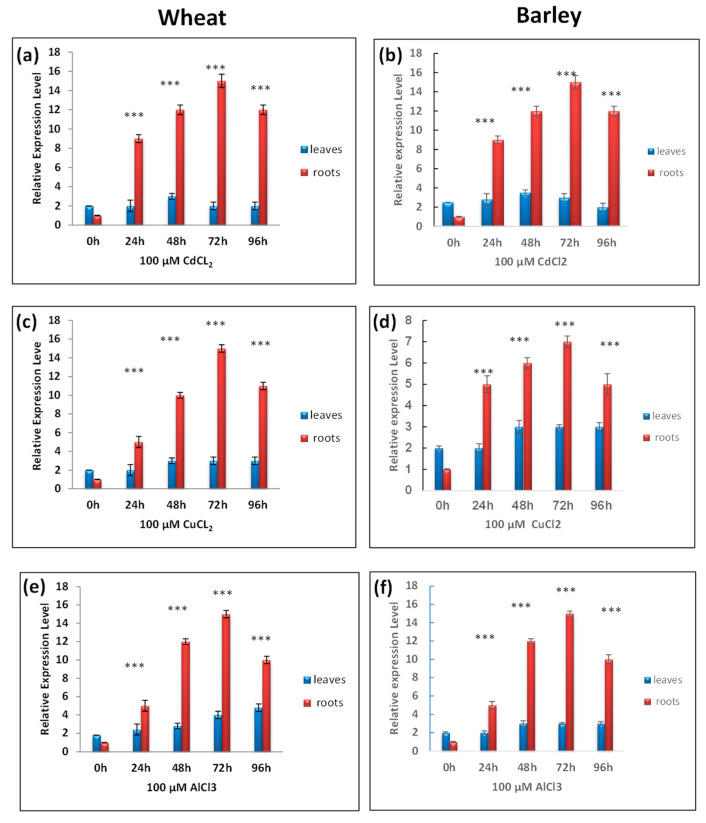
RT-qPCR expression analysis of TtCAT1 and HvCAT1 genes under different metallic stresses: (**a**,**b**) CdCL_2_, (**c**,**d**) CuCL_2_*, and* (**e**,**f**) AlCl_3_ in both wheat and barley species. The red bars represent the expression levels of the CAT-1 gene in wheat, and the blue bars represent the expression levels of the CAT-1 gene in barley. *** Indicates values significantly different from the control. Statistical significance was assessed by applying Student’s *t*-test at *p* < 0.01.

The expression patterns of TtCAT1 and HvCAT1 were also studied in response to metallic stress. In the presence of 100 µM of CdCl_2_, the expression levels of TtCAT1 and HvCAT1 increased slightly in leaves with a maximum-fold induction after 48 h of stress application, and the expression levels started to decrease after 72 h and 96 h, respectively. In roots, the expression of these genes was strongly induced in such stress conditions with a maximum-fold induction after 72 h of stress application in both species (Figure 8a,b). The same expression pattern was observed in the presence of CuCl_2_ stress with an expression level more pronounced in barley leaves compared with wheat (Figure 8cd). Interestingly, the expression level did not decline after 96 h of stress application in both species. In presence of AlCl_3_, the expressions of TtCAT1 and HvCAT1 increased gradually (in leaves) with time and reaches their maximum after 96 h of stress application. In roots, the expression of CAT1 genes started to increase after 24 h of stress application and reaches its maximum after 72 h (Figure 8ef).

**Figure 9:**
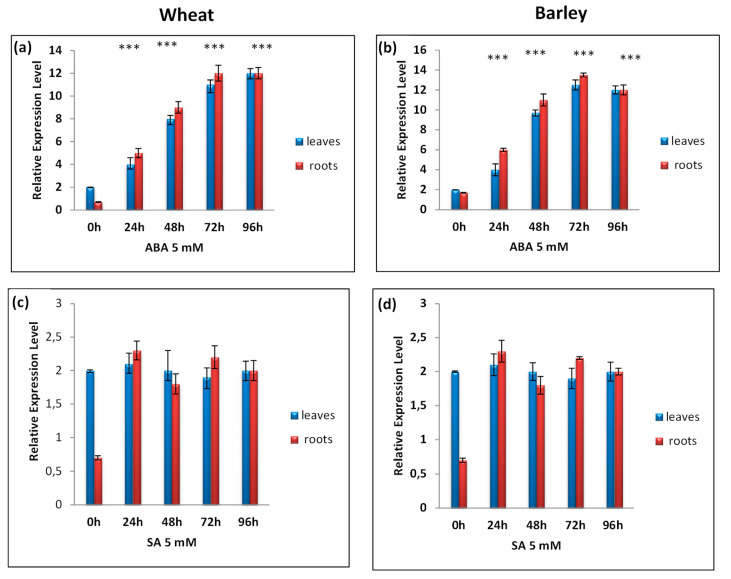
RT-qPCR expression analysis of TtCAT1 and HvCAT1 genes under hormonal stresses, ABA (**a**,**b**) *and* SA (**c**,**d**), in both wheat and barley species. The red bars represent the expression levels of the CAT-1 gene in wheat, and the blue bars represent the expression levels of the CAT-1 gene in barley. *** Indicates values significantly different from the control. Statistical significance was assessed by applying Student’s *t*-test at *p* < 0.01.

Finally, gene expression was also investigated in plants’ responses to hormonal stress. As shown in Figure 9, abscisic acid induced a rapid response in the CAT1 gene in both species after 24 h of stress application and continued to increase to reach the maximum after 72 h. In wheat, this expression remained stable, whereas in barley, the expression level of HvCAT1 started to decrease (Figure 9a,b). Together, these results confirm that catalase proteins that belong to subfamily I are implicated in plant responses to different abiotic stresses.

### 3.5. Changes in the TtCAT and HvCAT Activities under Different Abiotic Stresses

**Figure 10:**
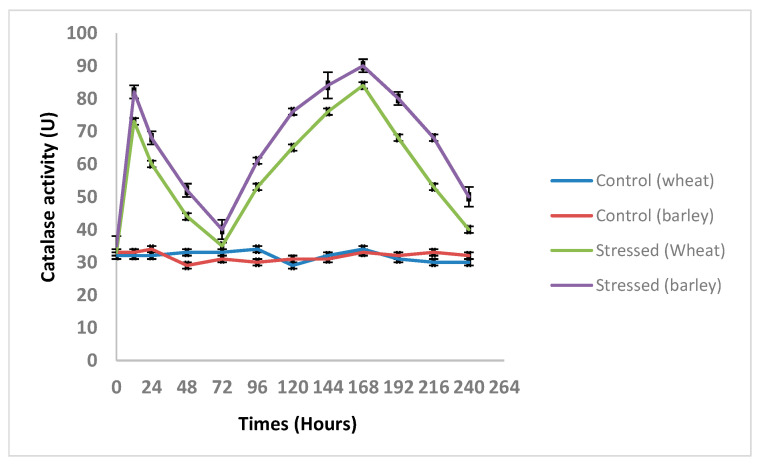
Catalase activity measured in barley and wheat plants under normal and salt stress conditions.

The responses of the TtCAT1 and HvCAT1 activities to salt stress were investigated in wheat (cv. Waha) and barley, respectively. Relative to the control, both catalase protein activities increased with the duration of treatment with the tested abiotic stresses. Interestingly, the patterns varied with the type of stress (Figure 10). Under NaCl treatment, CAT activity showed M”-type asymmetry (Figure 10): first, it increased (at 0.5 d) and then decreased after 1 day of exposure to the stress, followed by a gradual increase from 3 days to 7 days, with the highest value (1.8-fold as the control) at day 7 (P b 0.05), and finally declined.

## 4. Discussion

In plants, catalases are the most important scavenger proteins of H_2_O_2_. These toxic compounds could be generated during plant growth, normal metabolic processes, as well as environmental stress responses [46]. Plant catalases form a small gene family [11]. Regarding their importance in cells, many CAT genes have been isolated and characterized in dicotyledonous and monocotyledonous species. Plant catalases have been divided into three different classes, and different genes were identified in rice [47], durum wheat cv. Om Rabiaa [7], *T. monococcum* [8], *Triticum aestivum* [13], *Panax ginseng* [40], *Brassica napus* [14], cotton [10], and cucumber plants [11]. Thus, understanding the biological roles of CAT genes in monocotyledonous and especially in durum wheat and barley as well as the molecular mechanisms regulating their responses to stresses are crucial for the identification of new wheat varieties with enhanced resistances to different environmental constraints. In the present study, we isolated the full-length catalase genes TtCAT1 from the durum wheat cv. Waha and HvCAT1 from barley cultivated in Saudi Arabia. The Waha variety is characterized by short plants resistant to many fungal diseases [48] but sensitive to salt and cold stresses. The Waha variety is cultivated in many regions of the world such as North Africa and Saudi Arabia. The phylogenetic analysis revealed that TtCAT1 is close to the TaCAT1 and HvCAT1 catalase proteins which belong to class I. In addition, bioinformatic analyses indicated that TtCAT1 harbors all the conserved domains such as the N-terminal active site (54–70) with conservation of the catalytic residue at position 65, the catalase core domain (IPR011614; position 18–401), the catalase immune-responsive domain (IPR010582; 423–486), and the catalase heme binding sites (344–352). Structure analysis showed that all these domains are identified in bread wheat, *Brachypodium*, and barley except for durum wheat cv. Om Rabiaa; TdCAT1 [7,8]. Thus, we can conclude that all these domains are essential for catalase activity as they are highly conserved among species. In addition, the TtCAT1 protein harbors a conserved calmodulin binding domain located at the C-terminal portion of the protein. Moreover, the 3D structure of TtCAT1 was performed using *Bacillus pumilus* catalase as a template [41]. This analysis showed that TtCAT1 is a tetrameric protein which is typical for other catalases such as TaCAT1 [49], TmCAT1 [8], and TdCAT1 [7]. Typical catalases contain the heme group, implicated in the catalytic activity of TtCAT1. This group is located between the internal walls of the beta barrel and several helices, with conservation of aa forming the heme group: His 65, Ser l04, Asn 138, Arg 344, and Tyr 348 [7,8,50]).

TtCAT1 harbors a putative peroxisomal localization target PTS1 in its C-terminal part. Using in silico analysis, different databases demonstrated that this domain confers a peroxisomal localization to TtCAT1. The same domain was identified in different catalases from plants such as *P. ginseng* [40], pumpkin catalase (Cat1) [41], *T. turgidum* subsp. durum cv. *Om Rabiaa* [7], and *T. monococcum* [8]. Recently, it has been demonstrated that this domain is crucial for CAT1 localization as its deletion suppresses the peroxisomal localization of TmCAT1 [8]. Interestingly, the conserved three amino acids (PSI or PSM) found in all identified catalases are not important for catalase translocation into peroxisomes [51]. It has been shown that pumpkin catalase 1 interacts with the peroxisomal biogenesis factor peroxin 5 (PEX5) to facilitate its entry into the peroxisome [51]. In addition, the leucine amino acid, located at position 11 from the C-Terminal part of AtCAT2, is crucial for protein localization as the substitution of the leucine residue by a glycine inhibits the catalase import to peroxisome [52]. This localization may suggest that catalase could play a crucial role in photorespiration, hormone metabolism, lipid metabolism, and plant responses to surrounding stresses [53].

During recent years, S-nitrosylation has been considered as the most investigated protein post-translational modification (PTM). S-nitrosylation controls many processes such as hormone signaling in plants via a group of proteins called transnitrosylase [54]. In plants, Reactive oxygen species (ROS) and reactive nitrogen species (RNS) are controlled by S-nitrosylation (regulated via catalase proteins; [54]). In *Arabidopsis*, it has been shown that AtCAT3 has a transnitrosylase activity at Cys-10, and it is required for S-nitrosylation to regulate oxide signaling in *Arabidopsis* via the highly conserved residue Cyc-343 present in AtCAT3 and not AtCAt2 proteins [55]. In this work, we showed that TtCAT1 and HvCAT1 presented four and five nitrosylated sites, respectively (Supplemental Figure S3). C86, C230, C325, and C370 residues were conserved in both proteins, whereas HvCAT presented another site at C421. These results suggest that further investigations are needed to establish where TtCAT1 and HvCAT1 can be nitrosylated in wheat and barley, respectively. Mutations of these residues are needed to clarify the role of these residues in vivo during plant responses to different stimuli as well as during hormonal signaling and plant development. In fact, different studies revealed that S-nitrosylation is detected during plant responses to auxin signaling via the TIR1 protein in *A. thaliana* and *Triticum aestivum* L. [6,56]. BIN2 isolated from *Zea mays* was also shown to positively regulate brassinosteroid signaling in plants [6].

The presence of nitrosylated sites in catalase proteins was shown in bovine catalase, which presented one site at Cys 377 in the LGPNYLQIPVNCPYR motif and at Cys370 in sweet pepper (*Capsicum annuum*) [57]. Four cysteine residues were detected in the durum wheat (cv. Om Rabiaa) TdCAT1 sequence. The C86, C230, and C325 residues were conserved, but TdCAT1 presented a putative S-nitrosylated residue at the C470 position absent in TtCAT1. The three C86, C230, and C325 residues are also conserved in different monocotyledonous and dicotyledonous catalases [44].

In different plants, it has been shown that catalase genes play crucial roles in controlling plants’ responses to biotic and abiotic stresses [6,7,8,18,40]. In the Waha variety, we have no information about the molecular regulation during plants’ responses to abiotic stresses. In the Om Rabiaa variety, researchers demonstrated that TdCAT1 was upregulated by salt stress (with maximum induction after 3 days), PEG induced osmotic stress (with maximum induction after 2 days), H_2_O_2_ (oxidative stress with maximum induction after 24 h), and manganese (maximum induction after 6 days) [7]. In order to investigate the role of TtCAT1 in *Triticum turgidum* var Waha, its expression profile was analyzed in different tissues and under various environmental conditions. Under standard conditions, TtCAT1 is highly expressed in leaves (Figure 6a) as previously shown for other plants such as AtCAT2 in *Arabidopsis* [18], PgCAT1 in *Panax ginseng* [40], TdCAT1 in *Triticum turgidum* subsp durum cv. Om Rabiaa [7], and TmCAT1 in *Triticum monococcum* [8]. The same effect was observed in barley, where HvCAT1 was slightly induced in roots but highly induced in leaves under normal conditions (Figure 6b). Interestingly, stress application enhances TtCAT1 expression with differential expression patterns between organs. The same effect was also reported for TmCAT1 [8].

## 5. Conclusions

In conclusion, two peroxisomal catalase encoding genes (TtCAT1 from durum wheat and HvCAT1 from barley) were identified and cloned. TtCAT and HvCAT1 proteins are 492 aa in length and have a predicted molecular weight of 56.8 kDa. Moreover, the presence of the conserved domains, such as the catalytic active site, the peroxisomal targeting sequence, the calmodulin binding domain, and the heme binding motif, confirmed that it is a putative peroxisomal catalase. Using qRT-PCR analysis, we showed that TtCAT1 and HvCAT1 genes were expressed relatively high in leaves and stems comparing with roots in standard conditions. TtCAT1 and HvCAT1 were induced rapidly in 10-day-old seedlings using various stimuli such as osmotic agents (mannitol, PEG, and NaCl treatment), heavy metals (copper and cadmium treatments), and chilling stress. The positive responses of TtCAT1 and HvCAT1 to different abiotic stresses suggest that these catalases may play crucial roles in stress responses.

## Figures and Tables

**Table 1:** Comparison between TtCAT1 and HvCAT1 sequences with different isolated plant catalases using the ProtParam tool (http://web.expasy.org/protparam/, accessed on 2 November 2022).

Protein	Total Number of Negatively Charged Residues (Asp + Glu):	Total Number of Positively Charged Residues (Arg + Lys):	Grand Average of Hydropathicity (GRAVY):	MW	Number of aa
TtCAT1	63	58	−0.595	56807.99	492
HvCAT1	63	58	−0.591	56567.65	492
TdCat1	65	58	−0.600	56768.91	492
TaCat1	63	58	−0.595	56807.99	492
AetCAT1	63	58	−0.598	56723.87	492
TmCAT1	62	58	−0.590	56661.85	492
BdCat1	61	58	−0.608	56746.93	492
AtCat1	62	58	−0.574	56917.27	492
TdcCAT1	63	58	−0.599	56767.93	492
PgCAT1	61	57	−0.575	56766.98	492
SaCAT1	61	59	−0.548	56921.49	492
GhCAT1	61	56	−0.537	56855.20	492
LjCAT1	61	57	−0.547	56893.19	492
EsCAT1	59	58	−0.522	56689.18	492
GrCAT1	61	57	−0.538	56817.20	492
AiCAT1	61	58	−0.562	56955.22	492

**Table 2:** Secondary structure (2D) analysis of plant catalases using the SOPMA program.

Protein	α-Helices	β-Turns	Random Coils	Extended Strands
TtCAT1	140	27	242	83
HvCAT1	136	26	258	72
TdCat1	135	27	256	74
TaCat1	140	27	242	83
AetCAT1	127	30	258	77
TmCAT1	137	30	249	76
BdCat1	134	29	257	72
AtCat1	126	31	260	75
TdcCAT1	131	28	258	75
PgCAT1	138	29	250	75
SaCAT1	135	27	253	77
GhCAT1	139	29	249	75
LjCAT1	130	25	262	75
EsCAT1	137	28	255	72
GrCAT1	141	29	248	74
AiCAT1	134	32	247	79

## Data Availability

Data is contained within the article and Supplementary Material.

## Supplementary Materials

The following supporting information can be downloaded at: https://www.mdpi.com/article/10.3390/antiox11112208/s1, Figure S1: Protein sequence alignment of (a) catalase proximal active site signature domain and (b) catalase proximal heme–ligand signature of TtCAT1 with other plant catalase proteins using the ClustalW database. Figure S2: In silico localization of (a) HvCAT1 and (b) TtCAT1 proteins. Figure S3: Identification of potential phosphorylation sites in (a) TtCAT1 and (b) HvCAT1 proteins using the NetPhos 3.1 database. Figure S4: Identification of putative S-nitrosylation sites in TtCAT1 (a) and HvCAT1 (b) proteins using the GPS-NSO.1 database.
