# Supplementary material for: Identification and Expression Profiling of Two Saudi Arabia Catalase Genes from Wheat and Barley in Response to Abiotic and Hormonal Stresses
Source: Antioxidants (Basel). 2022 Nov 8;11(11):2208. doi: 10.3390/antiox11112208 (PMC9686680; doi:10.3390/antiox11112208)
Supplement: Supplementary file 1 [file antioxidants-11-02208-s001.zip › antioxidants-1994150-supplementary-proof done.pdf]

(a)

```
EsCAT1       DYQLVEIIANFDRERIPERVVHARGASAKGFFEVTHD
PgCAT1       DYQLVEKIANFDRERIPERVVHARGASAKGFFEVTHD
AtCat1       DYHLVEKLANFDRERIPERVVHARGASAKGFFEVTHD
SaCAT1       DYHLVEKLANFDRERIPERVVHARGASAKGFFEVTHD
GhCAT1       DYHLVEKLANFDRERIPERVVHARGASAKGFFEVTHD
LjCAT1       DYHLVEKLANFDRERIPERVVHARGASAKGFFEVTHD
GrCAT1       DYHLVEKLANFDRERIPERVVHARGASAKGFFEVTHD
AiCAT1       DYHLVEKLANFDRERIPERVVHARGASAKGFFEVTHD
BdCat1       DYHLVEKIANFDRERIPERVVHARGASAKGFFEVTHD
TtCAT1       DYHLVEKIADFDRERIPERVVHARGATAKGFFEVTHD
TaCAT1       DYHLVEKIADFDRERIPERVVHARGATAKGFFEVTHD
HvCAT1:      DYHLVEKIADFDRERIPERVVHARGASAKGFFEVTHD
TdCat1       DYHLVEKIADFDRERIPERVVHARGASAKGFFEVTHD
AetCAT1      DYHLVEKIADFDRERIPERVVHARGASAKGFFEVTHD
TmCAT1       DYHLVEKIADFDRERIPERVVHARGASAKGFFEVTHD
TdcCAT1      DYHLVEKIADFDRERIPERVVHARGASAKGFFEVTHD
             **:*** :*:****************:**********
```

(b)

```
LjCAT1       YSDDKMLQTRIFSYADSQRHRLGPNYLQ-
TdCat1       YSDDKLLQTRIFSYSETQRHRLGANYLLL
TtCAT1       YSDDKLLQTRIFSYSDTQRHRLGPNYLL-
TaCAT1       YSDDKLLQTRIFSYSDTQRHRLGPNYLL-
AetCAT1      YSDDKLLQTRIFSYSDTQRHRLGPNYLL-
TmCAT1       YSDDKLLQTRIFSYSDTQRHRLGPNYLL-
HvCAT1       YSDDKLLQTRIFSYSDTQRHRLGPNYLL-
BdCat1       YSDDKLLQTRIFSYSDTQRHRLGPNYLL-
TdcCAT1      YSDDKLLQTRIFSYSDTQRHRLGPNYLL-
GhCAT1       YSDDKLLQTRIFSYSDTQRHRLGPNYLQ-
GrCAT1       YSDDKLLQTRIFSYSDTQRHRLGPNYLQ-
PgCAT1       YSDDKLLQSRIFSYADTQRHRLGPNYLQ-
AtCat1       YSDDKLLQTRVFSYADTQRHRLGPNYLQ-
SaCAT1       YSDDKLLQTRIFSYADTQRHRLGPNYLQ-
EsCAT1       YSDDKLLQTRIFSYADTQRHRLGPNYLQ-
AiCAT1       YSDDKLLQTRIFSYADTQRHRLGPNYLQ-
             *****:**:*:***:::****** ***
```

Figure S1: Protein sequence alignment of Catalase proximal active site signature domain (a) and Catalase proximal heme-ligand signature (b) of TtCAT1 with other plant catalase proteins using clusterW database.

SeqID: HvCAT1:

CELLO predictor:

Localization Prediction

Localization	Score
Extracellular	0.079
Plasmamembrane	0.032
Cytoplasmic	0.243
Cytoskeletal	0.021
ER	0.042
Golgi	0.013
Lysosomal	0.037
Mitochondrial	0.362
Chloroplast	0.115
**Peroxisomal**	**3.893**
Vacuole	0.021
Nuclear	0.143

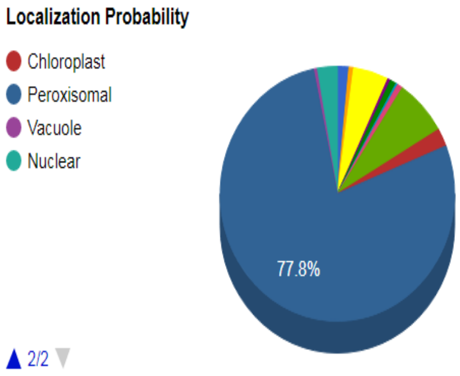

SeqID: TtCAT1

CELLO predictor:

Localization Prediction

Localization	Score
Extracellular	0.080
Plasmamembrane	0.057
Cytoplasmic	0.348
Cytoskeletal	0.022
ER	0.062
Golgi	0.014
Lysosomal	0.043
Mitochondrial	0.348
Chloroplast	0.129
**Peroxisomal**	**3.735**
Vacuole	0.024
Nuclear	0.136

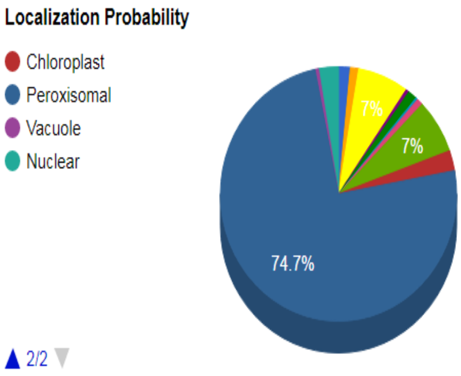

Figure S2: In silico localization of (a) HvCAT1 and (b) TtCAT1 proteins.

(a)

```
MDPYKYRPSSSFNAPMWSTNSGAPVWNNDNSLTVGSRGPILLEDYHLVEK   #    50
IADFDRERIPERVVHARGATAKGFFEVTHDVSHLTCADFLRAPGVQTPVI   #   100
VRFSTVIHERGSPETLRDPRGFAIKFYTREGNWDLVGNNFPVFFIRDGMK   #   150
FPDMVHALKPNPKTHIQENWRILDFFSHHPESLHMFTFLFDDIGVPADYR   #   200
HMDGSGVNTYTLVNRAGKAHYVKFHWKPTCGVKSLLEEEAVTVGGTNHSH   #   250
ATKDLTDSIAAGNYPEWTFYIQTIDPDYEERFDFDPLDVTKTWPEDVVPL   #   300
QPVGRLVLNRNIDNFFSENEQLAFCPGIIVPGVYYSDDKLLQTRIFSYSD   #   350
TQRHRLGPNYLLLPANAPKCSHHNNHYDGLMNFMHRDEEVDYFPSRFDPA   #   400
KHAPRYPIPSRTLNGRREKMVIEKENNFKQPGERYRSMDPARQERFINRW   #   450
IDALSDPRLTHEIKAIWLSYWSQADKSLGQKLASRLSSKPSM           #   500
%1  ...Y....SSS.......T...........S.T.................   #    50
%1  ...................T.......T..................T...   #   100
%1  ...S.......S..T...................................   #   150
%1  ...............................S..................   #   200
%1  ....S...............Y.......T....S................   #   250
%1  .T...T.............Y..T....Y.............T........   #   300
%1  ................S.........................T...S...   #   350
%1  T........................................Y........   #   400
%1  .....Y............................Y.S.............   #   450
%1  ....S....T........S..S....S......S..SS....
```

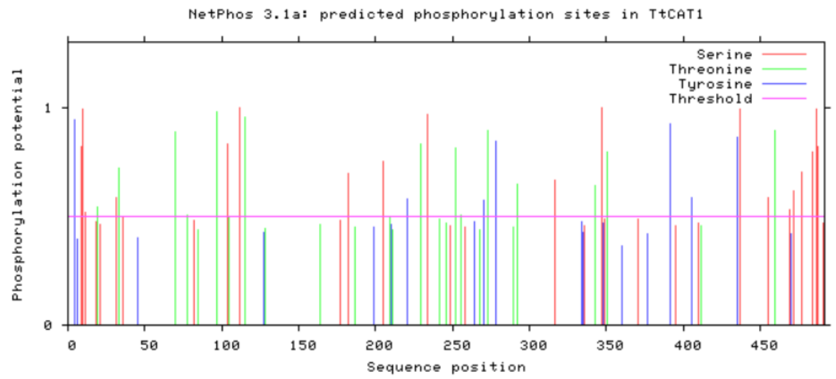

(b)

```
#
MDPYKHRPSSSFNSPMWSTNSGAPVWNNDNSLTVGSRGPILLEDYHLVEK   #    50
IADFDRERIPERVVHARGASAKGFFEVTHDVSHLTCADFLRAPGVQTPVI   #   100
VRFSTVIHERGSPETLRDPRGFAIKFYTREGNWDLVGNNFPVFFIRDGMK   #   150
FPDMVHALKPNPKTHIQENWRVLDFFSHHPESLHMFTFLFDDIGVPADYR   #   200
HMDGSGVNTYTLVNRAGKAHYVKFHWKPTCGVKSLLEDEAVTVGGTNHSH   #   250
ATKDLTDSIAAGNYPEWTFYIQTIDPDHEDRFDFDPLDVTKTWPEDVVPL   #   300
QPVGRLVLNRNIDNFFAENEQLAFCPGIIVPGVYYSDDKLLQTRIFSYSD   #   350
TQRHRLGPNYLLLPANAPKCSHHNNHYDGLMNFMHRDEEVDYFPSRFDPA   #   400
KHAPRYPIPARALNGRREKACIDKENNFKQPGERYRSMDPARQERFINRW   #   450
IDALSDPRLTHEIKAIWLSYWSQADKSLGQKLASRLSAKPSM           #   500
%1  ...Y....SS...S................S.T.................   #    50
%1  ...................S.......T..................T...   #   100
%1  ...S.......S..T...................................   #   150
%1  ...............................S..................   #   200
%1  ....S...............Y.......T....S.......T........   #   250
%1  .T...T.............Y..T..................T........   #   300
%1  ..........................................T...S...   #   350
%1  T........................................Y........   #   400
%1  .....Y............................Y.S.............   #   450
%1  ....S....T........S..S....S......S..S.....
```

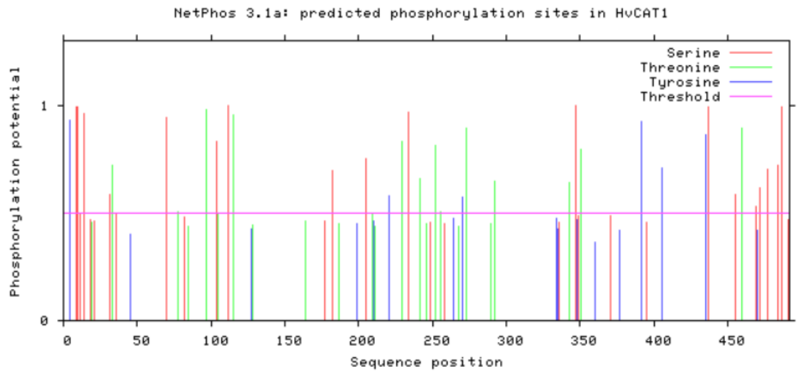

Figure S3: Identification of potential phosphorylation sites in (a) TtCAT1 and (b) HvCAT1 proteins using Netphos 3.1 database.

(a)

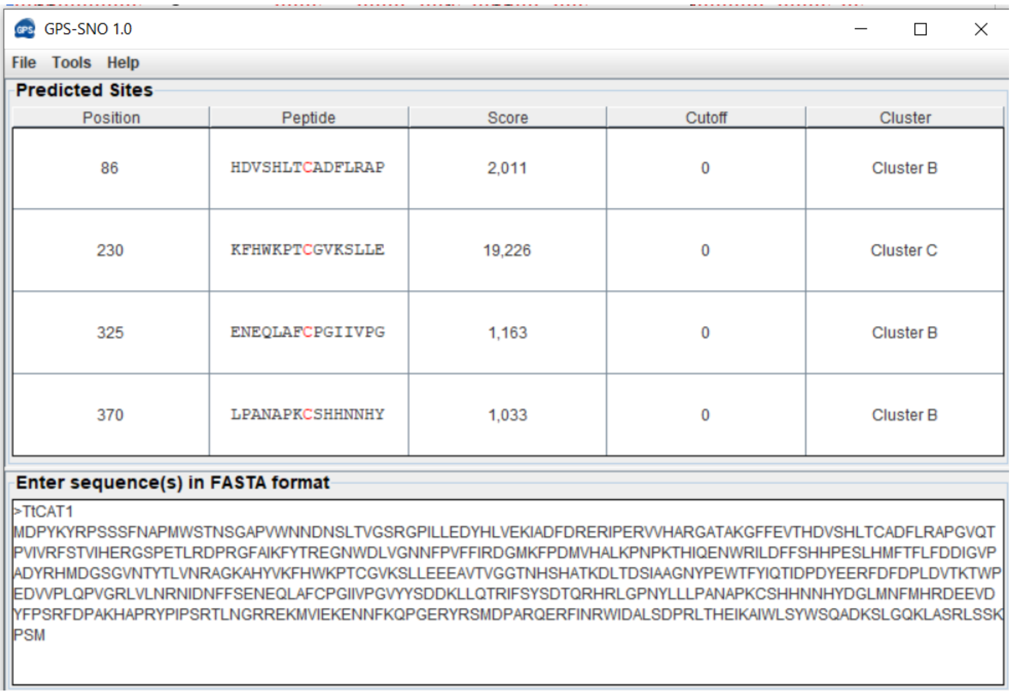

Position	Peptide	Score	Cutoff	Cluster
86	HDVSHLTCADFLRAP	2,011	0	Cluster B
230	KFHWKPTCGVKSLLE	19,226	0	Cluster C
325	ENEQLAFCPGIIVPG	1,163	0	Cluster B
370	LPANAPKCSHHNNHY	1,033	0	Cluster B

(b)

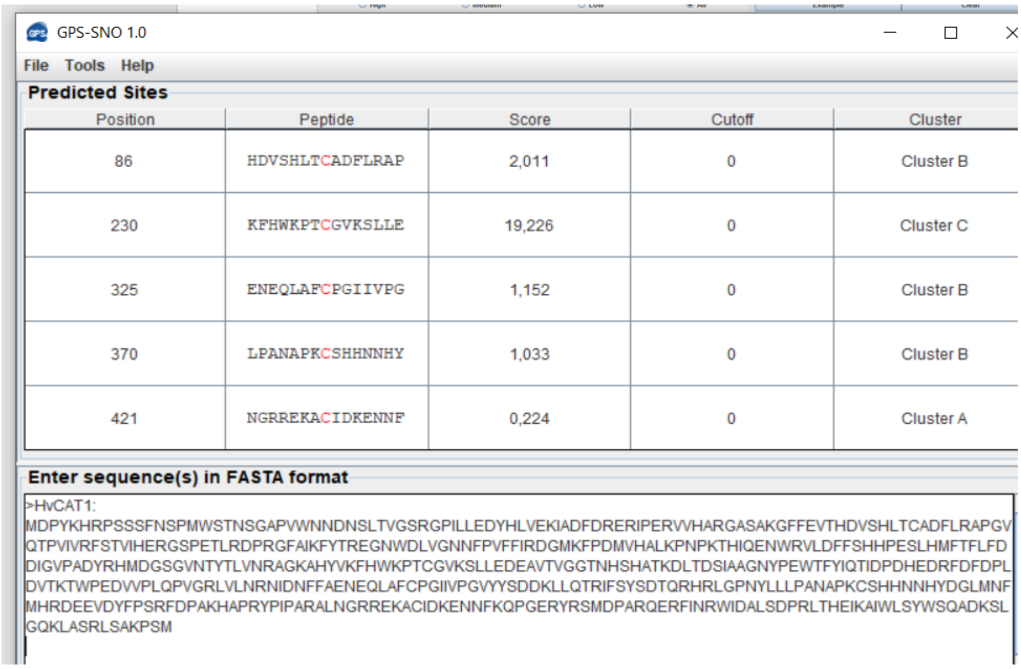

Position	Peptide	Score	Cutoff	Cluster
86	HDVSHLTCADFLRAP	2,011	0	Cluster B
230	KFHWKPTCGVKSLLE	19,226	0	Cluster C
325	ENEQLAFCPGIIVPG	1,152	0	Cluster B
370	LPANAPKCSHHNNHY	1,033	0	Cluster B
421	NGRREKACIDKENNF	0,224	0	Cluster A

Figure S4: Identification of putative S-nitrosylation sites in TtCAT1 (a) and HvCAT1 (b) proteins using GPS-NSO.1 database.
